# Spatial Distribution of Bednet Coverage under Routine Distribution through the Public Health Sector in a Rural District in Kenya

**DOI:** 10.1371/journal.pone.0025949

**Published:** 2011-10-12

**Authors:** Wendy Prudhomme O'Meara, Nathan Smith, Emmanuel Ekal, Donald Cole, Samson Ndege

**Affiliations:** 1 Department of Medicine, Duke University School of Medicine, Durham, North Carolina, United States of America; 2 Duke Global Health Institute, Durham, North Carolina, United States of America; 3 Department of Epidemiology and Nutrition, Moi University School of Public Health, Eldoret, Kenya; 4 United States Agency for International Development-Academic Model Providing Access to Healthcare Partnership, Eldoret, Kenya; 5 Ministry of Public Health and Sanitation, Nairobi, Kenya; 6 Division of Global Health, Dalla Lana School of Public Health, University of Toronto, Toronto, Ontario, Canada; Kenya Medical Research Institute - Wellcome Trust Research Programme, Kenya

## Abstract

Insecticide-treated nets (ITNs) are one of the most important and cost-effective tools for malaria control. Maximizing individual and community benefit from ITNs requires high population-based coverage. Several mechanisms are used to distribute ITNs, including health facility-based targeted distribution to high-risk groups; community-based mass distribution; social marketing with or without private sector subsidies; and integrating ITN delivery with other public health interventions. The objective of this analysis is to describe bednet coverage in a district in western Kenya where the primary mechanism for distribution is to pregnant women and infants who attend antenatal and immunization clinics. We use data from a population-based census to examine the extent of, and factors correlated with, ownership of bednets. We use both multivariable logistic regression and spatial techniques to explore the relationship between household bednet ownership and sociodemographic and geographic variables. We show that only 21% of households own any bednets, far lower than the national average, and that ownership is not significantly higher amongst pregnant women attending antenatal clinic. We also show that coverage is spatially heterogeneous with less than 2% of the population residing in zones with adequate coverage to experience indirect effects of ITN protection.

## Introduction

Insecticide treated bednets (ITNs) are one of the most cost-effective and widely used malaria interventions [1], [2]. Between 2006–2008, more than 140 million nets were manufactured and delivered for distribution in sub-Saharan Africa [3].

Maximum effectiveness of ITNs is achieved when a high percentage of individuals in a geographic area are using ITNs. It is estimated that substantial protective indirect effects are seen with roughly 50% or greater coverage of entire populations[4], [5], [6]. Strategies for distributing ITNs differ between countries and between programs and they show a high degree of variability in coverage of households and high-risk groups [7], [8]. Generally, unsubsidized ITNs provided through the private retail sector produces the lowest coverage with significant differences between socioeconomics groups. Free, community-based mass distribution campaigns have been shown to sharply increase bednet coverage and reduce inequities in bednet ownership across socioeconomic strata [2], [7], [8], [9], [10], [11]. Although mass distribution campaigns are effective, they are also expensive and require repeated campaigns to replace old, damaged, or expired nets. Many programs provide free or partially subsidized ITNs to high-risk groups through routine contact with government health services, particularly antenatal clinics (ANC) and immunization clinics. Still other countries have relied on social marketing of ITNs, and have scaled-up distribution through both the health sector and the private retail sector, usually involving a small co-pay [9]. Such cost-sharing schemes with private and public sector subsidies have sustained high coverage [10]. Other studies suggest that a mix of distribution mechanisms can both achieve and maintain high and equitable coverage [9], [11], [12].

Delivery of ITNs, either free or partially subsidized, through the government health sector remains the most common avenue for ITN distribution in most countries in sub-Saharan Africa. It is the most logistically straightforward, least expensive, and targets those who bear the greatest burden of disease. In Kenya, the main channel for ITN distribution is through the government health facilities, particularly to pregnant women who attend antenatal clinics and infants who are seen in the immunization clinics. In some areas, mass distribution campaigns or social marketing channels have been used, but these approaches have been limited in geographic scope and frequency [13].

The purpose of this analysis was to describe the impact of routine distribution of bednets targeted to high-risk groups through government health facilities on household-level ownership, and explore some determinants of ownership. We present bednet ownership data from a complete census conducted in a rural district in western Kenya. We explore spatial heterogeneity of bednet ownership at the household level and compare coverage in targeted groups. We evaluate whether targeting high-risk groups attending health facilities is achieving the required coverage in the targeted groups as well as the population as a whole.

## Methods

The bednet study was conducted from retrospective analysis of data collected during implementation of a large, home-based public health program, augmented with data collected from health facilities in the implementation area. Study site, study population and data collection procedures are described below.

### Study site

Bungoma East district is located in Western Province, Kenya about 50 km from the border with Uganda. It is divided into 23 administrative units called sublocations. There is a river that borders the district to the east and another river that transects the northwestern part of the district. Residents are primarily subsistence farmers, although there are two large sugar plantations which employ a large number of day-laborers from the surrounding communities. The major road between Nairobi and Uganda runs through the middle of the district. There is a small town center. The population is estimated to be just over 200,000 people. Malaria transmission is year-round with a seasonal peak following the rains in March to May. Annual EIR is 29 and more than 60% of children were parasitemic in cross sectional surveys during the rainy season [14].

Government-owned health facilities are categorized from level 2–6. Level 2 is used to describe dispensaries, level 3 refers to health centres which typically have laboratory capacity, more staff, and larger formularies than dispensaries. Level 4–6 facilities are hospitals at the district, provincial or national level with in-patient services and increasing capacity at each level. The population in the study area is served by 21 government-owned health facilities, including a level-4 district hospital, 3 health centres and 17 dispensaries. There is also a mission-run hospital in the northern part of the district and three mission-run dispensaries.

### Routine facility-based data

Insecticide-treated bednets are distributed to pregnant women attending public health facilities for antenatal care and to children less than one year of age attending immunization or well-child clinics in keeping with Government of Kenya, Ministry of Public Health & Sanitation guidelines. There have been no community-based ITN distribution programs in the district in at least the last five years. The number of ITNs distributed through the public health facilities in the two years preceding data collection was recorded from routine records kept by the District Health Management Team.

### Population census data collection

Household data were collected as part of a large public health campaign initiated to identify HIV-infected individuals. This Home-based Counseling and Testing (HCT) campaign was undertaken by the Academic Model Providing Access to Healthcare (AMPATH) in Bungoma East District between July 2009 and April 2010. The program is described in detail elsewhere [15]. Briefly, all households in the district were visited to offer counseling and testing for HIV. Data were collected using Palm T|X PDA devices (Palm Inc®, California, USA). Standardized information was entered into data-collection forms programmed with Pendragon Forms Software (DDH Software, Inc®, Florida, USA). The total number of individuals resident in the household was recorded and all individuals older than 13 years were offered testing. All children less than five years were screened for immunization. Other data collected included individual demographic data, household asset information, HIV testing history and outcome, bednet ownership and GPS coordinates of the household via direct cable link to an external e-Trex GPS device (Garmin®, Kansas, USA). Pregnant women were identified and asked about attendance at the antenatal clinic. Data were collected from 96% of households in the district.

We refer to any nets reported in the household as ‘bednets’ because information about bednet retreatment and long-lasting insecticide treated nets was not collected. We define bednet ‘coverage’ as household ownership of at least one bednet.

A database of health facilities in Kenya including GPS coordinates was compiled and provided by researchers at KEMRI-Wellcome Trust-Nairobi [16]. The database was augmented with additional mapping within Bungoma East District using the handheld e-Trex GPS devices. All facilities were categorized according to level of service – dispensaries, health centres, and hospitals. Other geographic features, including major town centers and all roads (both paved and unpaved) that were accessible in a four-wheel drive vehicle were also mapped. GPS coordinates were uploaded and imported into a database of geographic features using DNRGarmin GPS application (Minnesota Department of Natural Resources, Minnesota, USA). Data for administrative boundaries and rivers were obtained from the Data Exchange Platform for the Horn of Africa (DEPHA) (United Nations, URL: http://www.depha.org), Africover (Food and Agricultural Organization [FAO] of the United Nations, URL: http://www.africover.org), and the World Resources Institute (URL: http://www.wri.org/publication/content/9291). All data was imported into ArcInfo v10.0 (Esri, California, USA).

### Data analysis

Sublocations were divided into urban and rural by comparison with Africover landmaps and knowledge of the local area. Only one of 23 administrative sublocations was classified as urban (∼3,900 households). All non-spatial data analysis was done in Stata v10. Chi-square tests were used for pair-wise comparisons of bednet ownership by household characteristics. Multivariable regression models were stratified into urban and rural sublocations. Logistic regression was used to explore the relationship between bednet coverage in urban populations and sociodemographic and geographic variables. For rural areas, mixed effects logistic regression models were used with a random effect for sublocation to account for unobserved differences between sublocations. The random effects were captured as random intercepts for each sublocation. The model with the random effects term fit significantly better than the model without. Coefficients are reported as odds ratios. An independent variable was considered to have a significant correlation with bednet ownership if the p-value was <0.05.

Descriptive spatial analysis was done using ArcInfo v10.0 (mapping), R GUI v1.4 for Mac OSX (cluster analysis), and ArcView 3.2 with the Nearest Feature (NearFeat) v3.8b extension (Jenness Enterprises). Ripley's K-function, K(d), was used to evaluate clustering of households without nets compared to households owning bednets. K-function values were calculated between 50 m and 1500 m in 50 meter increments and underestimation of unobserved neighbors near the edge of the study boundary was corrected for by using a border correction method. Household point patterns were tested against complete spatial randomness using 19 permutations of random point placement, yielding >90% confidence envelope. To compare patterns of clustering (households without bednets versus households owning bednets), the difference between the calculated K-function values was plotted against distance (d).

The NearFeat extension was used to calculate the Euclidean distance between features, including distance to the nearest health facility, type of nearest facility, and nearest mapped road.

Kernel density estimation was used to calculate the density of features within a defined area. A kernel estimation surface, based on the quadratic kernel function and a defined radius of 800 meters, was used to estimate the density of households around each 50 meter by 50 meter area (cell) across the study area. This was repeated to calculate the density of households with bednets for each 50 by 50 meter cell. The ratio of households with bednets to all households was calculated across all 50 meter by 50 meter areas within the study area. Areas with less than 20 households per 800 sq. meters were excluded to limit edge effects. Using ArcInfo v10.0, a raster image of the study area was generated using the values of the estimated ratio of households with bednets to total households for each 50 meter by 50 meter cell. Changing the kernel radius between 400–1600 meters did not significantly change the estimate of percent of households at each level of coverage. 800 meters was chosen to represent a neighborhood and corresponds with approximate vector ranges.

To estimate the neighborhood bednet coverage at each individual household location, the raster image values were extracted at each household location. Using the resulting bednet coverage values at each household location, the percent of households within certain coverage levels was calculated.

### Ethical approval

HCT is a home-based public health initiative. All participants gave voluntary informed consent for HIV testing. Consent was obtained verbally prior to data collection or any test being conducted. In the case of children less than 18, parental/guardian consent was obtained. In the context of a community health initiative, written consent was not considered appropriate. Verbal consent is considered the norm for most clinical care procedures and activities in our region. Documentation of verbal informed consent was collected by recording who had accepted household entry and testing.

The Institutional Review and Ethics Committee at Moi University and Moi Teaching and Referral Hospital in Eldoret, Kenya and Duke University Institutional Review Board approved the use of de-identified data from this program for analysis and publication.

## Results

### Bednet Distribution through public health facilities

Bednets were distributed through ANC and immunization clinics at all health facilities. According to the Bungoma East District Ministry of Health, no community-based, mass distribution campaigns were conducted in at least the previous five years. In 2008, a total of 9,148 bednets were distributed; in 2009, a total of 11,662 bednets were distributed (Table 1).

**Table 1:** Numbers of ITNs distributed through public facilities in Bungoma East district, Kenya, 2008 and 2009.

Year		
	2008	2009
*ANC Clinics*	3,110	5,499
*U1 Immunization Clinics*	6,038	6,163
*Total*	9,148	11,662

### Household Bednet Ownership

A total of 44,753 households were visited and household characteristics were collected. Only 21% (n = 9,532) of all households reported owning at least one bednet. Seventy-two percent of households with any bednet reported owning only one net, 18% reported two nets and the rest reported owning between 3–10 bednets. The total number of bednets reported in the census was 13,230, about 64% of the number reported distributed through facilities. Among households with a pregnant woman (n = 2,988), 25% owned at least one bednet (Table 2). Among households with children under 5 years old (n = 23,645), 25% owned at least one bednet. Among all other households (n = 19,950), 17% owned at least one bednet.

**Table 2:** Bednet Ownership and Distribution within Bungoma East District.

	n	Percent with at least one bednet	p-value
*Households with Pregnant Women*	2,988	25%	p = 0.97
*Households with Pregnant women attending ANC*	1,711	25%	
*Households with Children Under 5*	23,645	24%	p<0.001
*Households without U5 or pregnant women*	19,950	17%	
*Urban households*	3,497	18%	p<0.001
*Rural households*	41,256	22%	
**Total households**	**44,753**	**21%**	

### Household Characteristics and Univariate Analysis

The average household membership was 4.4 persons and 53% of households had one or more children less than five years of age. The proportion of households owning land was 76%, with 52% of all households owning animals. The majority of households lived closest to a dispensary (65%), a quarter of households lived nearest to a hospital (24%), and 11% lived closest to a health center. Households owning a bednet lived an average 2.07 km (SD = 1.01) from the nearest health facility, and households with no bednets lived an average 2.12 km away (SD = 1.01).

Amongst households with pregnant women, women attending ANC were not more likely to be in a household with a bednet (Table 1; p = 0.97). Households with children under 5 years were more likely to have a bednet (p<0.0001). Among urban and rural areas, households in rural areas were more likely to own a bednet than urban areas (p<0.0001).

### Multivariate Analysis

To understand the relationship among sociodemographic characteristics and bednet ownership, multivariate regression was performed on household variables. The analysis was stratified by urban and rural households. Table 3 shows the odds ratios of bednet ownership for each independent variable included in the model. In both the rural and urban households, the presence of children under 5 years in the household increased the odds of bednet ownership (OR = 1.17 urban, OR = 1.22 rural, p<0.01). The presence of a pregnant woman also significantly increased the odds of bednet ownership, but whether the expectant mother was attending ANC did not affect bednet ownership.

**Table 3:** Factors associated with household possession of at least one bednet in multivariable logistic regression analysis, stratified by broad location.

n = 3,497	OR	OR			
URBAN	unadjusted	adjusted	p-value	95% CI	
Children <5	1.09 (0.98, 1.22)	1.17	0.01	1.04	1.31
Pregnant mother	1.60 (1.11, 2.28)	2.01	0.02	1.14	3.55
Pregnant mother attending ANC	1.57 (0.98, 2.52)	0.85	0.67	0.41	1.78
*Wealth indicators*					
Own any animals	1.48 (1.21, 1.82)	1.47	0.00	1.18	1.84
Own any land	1.30 (1.09, 1.55)	1.39	0.05	1.01	1.92
Total animals	1.07 (1.04, 1.12)	1.03	0.35	0.97	1.08
*Distance*					
To Dispensary	1.83 (1.50, 2.26)	1.30	0.04	1.01	1.68
To Any facility	0.80 (0.73, 0.87)	1.39	0.33	0.72	2.67
To Health Centre^a^					
To Road	0.33 (0.26, 0.43)	0.38	0.00	0.27	0.53
*Proximity* b					
Nearest facility is hospital	7.55 (4.12, 13.86)	2.90	0.01	1.28	6.56
Nearest facility is Health Centre	(omitted)^a^				
*Pseudo R^2^*	*0.056*				

n = 41,246	OR	OR			
RURAL	unadjusted	adjusted	p-value	95% CI	
Children <5	1.21 (1.18, 1.24)	1.21	0.00	1.18	1.24
Pregnant mother	1.22 (1.12, 1.34)	1.18	0.02	1.03	1.36
Pregnant mother attending ANC	1.22 (1.08, 1.36)	1.03	0.72	0.87	1.23
*Wealth indicators*					
Own any land	1.43 (1.34, 1.52)	1.26	0.00	1.18	1.35
Own any animals	1.37 (1.30, 1.43)	1.15	0.00	1.08	1.23
Total animals	1.04 (1.04, 1.05)	1.02	0.00	1.01	1.04
*Distance*					
To Dispensary	1.02 (1.00, 1.03)	1.02	0.32	0.98	1.06
To Any facility	0.96 (0.93, 0.98)	0.87	0.00	0.84	0.90
To Health Centre	0.98 (0.97, 0.99)	1.02	0.04	1.00	1.04
To Road	0.91 (0.90, 0.92)	1.09	0.00	1.06	1.13
*Proximity* b					
Nearest facility is hospital	0.80 (0.75, 0.85)	0.90	0.08	0.80	1.01
Nearest facility is Health Centre	1.66 (1.55, 1.77)	1.20	0.01	1.05	1.37
		*Random effects parameters*			
		Standard dev. of constant (95% CI)	0.55	(0.40	0.74)
		Standard error	0.086		

Data presented here are the odds ratio (OR), p-value, and 95% confidence intervals (CI) for the multivariable logistic regression stratified by urban versus rural households.

a Within Webuye town the health centre and hospital are less than 0.5 km apart. The distance and proximity variables were combined to consider these two facilities equal.

b Reference variable is nearest facility is dispensary.

Among urban households, wealth indicators (land ownership OR = 1.39, p = 0.05 and animal ownership OR = 1.47, p<0.001) were strongly associated with bednet ownership. Households closest to a hospital were nearly three times as likely to own a bednet (OR = 2.90, p = 0.01). As distance to the nearest road increased, the odds of owning a bednet significantly declined (OR = 0.38, p<0.001).

In rural households, the wealth indicators also significantly increased the odds of bednet ownership, but the effect was smaller than for urban households. The largest change in odds of bednet ownership for rural households was related to whether the nearest facility was a health centre (OR = 1.20, p = 0.01) and how far away the household was from any facility (OR = 0.87, p<0.001). Although the distance to the nearest health centre (OR = 1.02, p = 0.04) or the nearest road (OR = 1.09, p<0.001) were each significant, the effects were very small per kilometer. Nevertheless, the cumulative effects of distance may be substantial; 20% of rural households are located more than 3 km from a facility, giving an odds ratio of 0.66 for bednet ownership in these households.

**Figure 1:**
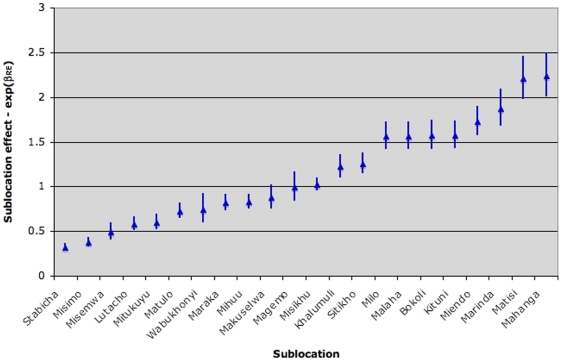
Exponential of the random effects (with 95% CI) for each sublocation for the mixed effects model. This plot shows the effect of sublocation of residence on bednet ownership. The random effects plot is the exponent of the random intercept for each sublocation. The exponent of the random effect can be thought of as the quantity that the exponent of the fixed effects intercept would be multiplied by to account for sublocation. So if exp(RE) = 1.5 then the exp(βo) would be multiplied by 1.5 for households in that sublocation. When exp(RE) = 1, that is the zero effect – location has no effect on the outcome. The plot shows that there is considerable heterogeneity between sublocations due to unobserved factors not captured in the model.

Significant differences in bednet ownership between sublocations were observed and these differences were not explained by the independent variables reported above. The heterogeneity is captured in the distribution of the random effects by sublocation estimated from the model. The spatial random effects with estimated 95% confidence intervals are plotted in Figure 1.

### Spatial Distribution

**Figure 2:**
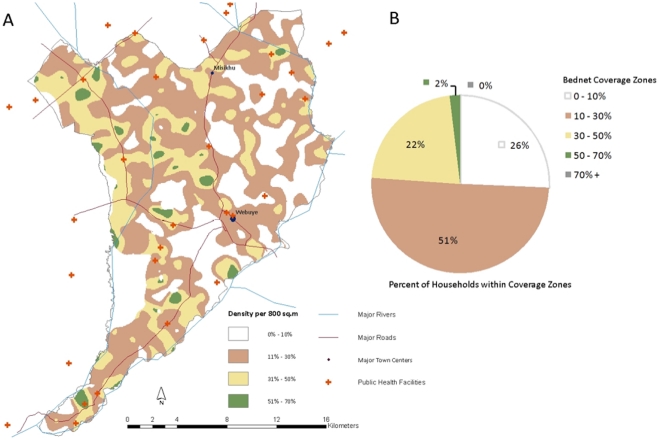
Spatial distribution of bednet coverage. (A) Map of household-level coverage raster. Areas with 0% to 10% community coverage are shown in white; areas with 11%–30% community coverage are shown in brown; areas with 31%–50% community coverage are shown in yellow; and areas with 51% –70% community coverage area shown in green. Major rivers, roads, town centers, and public health facilities are shown. (B) Percent of households within each coverage zones. Colors correspond to map.

Bednet coverage varied across the study area but was generally suboptimal; 77% of households lived in areas with 30% coverage or less (Figure 2). No areas were found to have coverage above 70%, and only 2% of households live in areas with greater than 50% coverage. A majority of the areas of higher coverage were seen to be northwest of Webuye town, with other areas to the southwest of the town center.

**Figure 3:**
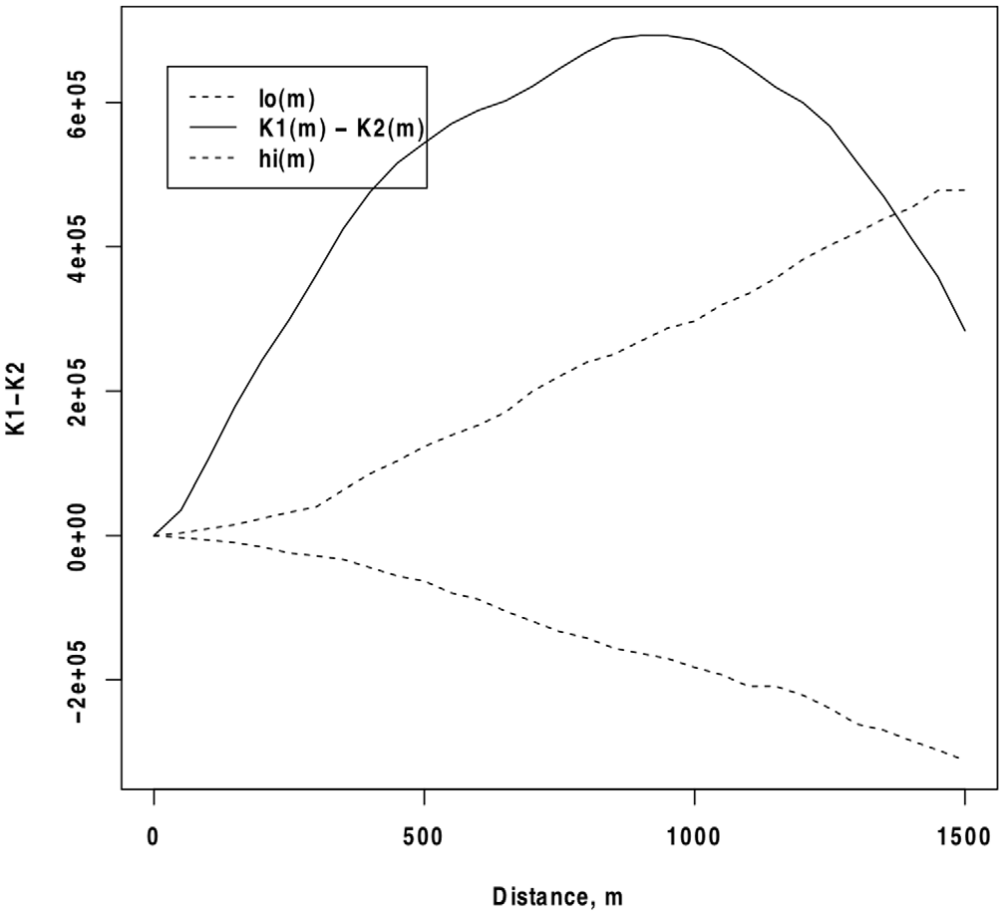
Cluster analysis of bednet coverage. The difference between K_1_(d), k-function for pattern of households owning at least one bednet, and K_2_(d), k-function for pattern of the underlying household distribution (solid line) and the confidence envelope (dashed lines) around the difference of expected distributions (zero line). Positive values indicate greater clustering of households owning at least one bednet in comparison to the underlying clustering of all households. Negative values indicate households owning at least one bednet have a more dispersed pattern than the underlying household distribution.

Computed K-function values showed that the distribution of all households and households owning bednets were significantly more clustered than would be expected from a random distribution of points (data not shown). The difference of the *K*-functions shows that households owning at least one bednet are significantly more clustered than households without bednets over a range of distances. Figure 3 shows the difference curve between the observed K values of households owning a bednet and households without bednets across the study area. Differences greater than zero indicate that households with a bednet are more clustered, or located near each other more often than households without bednets. Values less than zero would indicate that households with bednets are more dispersed than households without bednets. On average, households with bednets are more clustered than those without within a radius of between 50 m and 1200 m, with the greatest relative clustering seen at about 1000 m. At distances >1200 m, the difference is no longer greater than would be expected under spatial randomness.

## Discussion

The Kenya Division of Malaria Control promotes the implementation of insecticide treated bednets as a cornerstone of its malaria control strategy and employs several mechanisms to distribute ITNs. The primary distribution mechanism is through routine visits to government-owned health facilities, although mass distribution campaigns have been used. Countrywide, the percentage of households owning and using any type of bednet is 60%, while ownership of at least one ITN in the house is 56%. In Western Province, where our study area is located, 74% of households owned at least one bednet [17].

Bungoma East district uses targeted distribution of free ITNs through antenatal and immunization clinics. The data presented here show that household bednet ownership in Bungoma East district was far lower than both the national average and the provincial average. Only 21% of households reported owning at least one bednet. District-level data from Kenya shows considerable differences in bednet use between districts, ranging from less than 10% to more than 60% coverage [18]. Previous studies have shown similar population-level coverage in Kenya when ITNs were delivered through health facilities [19] so the low coverage observed here is not entirely unexpected. The low ITN coverage may be responsible for high reported morbidity; there were 49,700 episodes of clinical malaria reported in the district in 2010 (Ministry of Health data, E. Ekal), in a population of approximately 190,000 people.

Fifty-three percent of households in the study area had a child under 5 years and therefore should have recently been eligible for a free ITN, only 24% of these households owned a bednet. Ownership amongst pregnant women attending ANC compared to those not attending ANC was not significantly different despite the fact that women attending ANC were eligible for a free ITN *and* had recently visited the health facility. Recent contact with the ANC clinic should be correlated with high likelihood of bednet ownership and this may be a litmus test for the current availability and effectiveness of the facility-based distribution mechanisms. Our results differ from results seen in Zambia where distribution through ANC was paired with mass distribution[11].

When comparing the number of ITNs reported to be distributed in government health facilities in the two years preceding data collection with the number of households with children less than two years or pregnant women, there should not have been a shortfall of ITNs. These data suggest appropriate planning for supplies of ITNs but point to other factors limiting distribution. At least one other study has documented misuse of ITN distribution programs at the level of the health facility [20]. We cannot rule out leakage of public-sector ITNs into the retail sector, or informal charges levied by facility staff for ITNs despite Ministry of Health policy to distribute them free of charge. We can also not account for women residing outside the census area attending facilities within the census area, although we do not expect this to be a major factor in the discrepancies noted here. A recent study in Bioko Island of Equatorial Guinea showed a 30% decline in bednet ownership just one year after mass distribution [21]. This indicates that the actual lifespan and retention of bednets may be much lower than their predicted lifespan. This could partially account for the difference between the number of nets distributed and the number identified in the community. Another possible explanation is that new ITNs replace older ones rather than being added to the total number of nets in use.

Wealth indices correlate with bednet ownership even though bednets are provided for free in ANC and immunization clinics. This indicates that many of the bednets may have been purchased in the retail sector. Although wealth indicators may also reflect whether a family can afford to meet the costs of travel to a health facility and time away from daily activities, the observation that those who recently visited a facility (i.e. pregnant women attending ANC) were no more likely to have a bednet suggests that this was probably not a factor in accessing a bednet. Wealth has been shown to be a factor in bednet ownership in a number of other studies [22], [23], [24] and clear inverse relationships between poverty and uptake of ITNs has been shown [25].

The regression results revealed that distance to a facility is significant in predicting bednet ownership particularly in rural areas but is not as important as the type of facility nearest to each household. This seems to indicate that bednet distribution happens more effectively or more regularly at certain types of facilities (health centres may have a priority) or bednets have been distributed in only select facilities. While few studies have directly looked at bednet ownership and distance to public health facilities, studies elsewhere have looked at distance effects on utilization of health services and malaria morbidity, seeing clear reductions in malaria hospitalization with increasing physical access to primary health facilities [26], [27].

Population coverage of bednets is an important determinant of the impact of bednet programs [4], [5], [6]. Low coverage (less than 50% of individuals) has been shown to be associated with reduced community-level effects [4]. Our study revealed only 21% of households own a bednet, likely resulting in reduced community effects from these bednets. Targeted distribution of ITNs through health facilities generated significant heterogeneity of bednet coverage. Only 16% of households are in zones where the household coverage is at least 35–65% – a range estimated to provide both community and individual-level protection [6]. Only 2% of households were in zones with >50% coverage, a range that has been shown to provide protection from infection and anemia to non-ITN users [4]. Here we have only assessed household-level ownership and have not measured the proportion or household members sleeping under a bednet, a parameter that has been shown to be important in other studies [28]. Furthermore, household ownership (as we have defined “coverage”) is not equivalent to population coverage (fraction of the population owning or sleeping under a bednet).

The results presented here showing differences among targeted groups and the general population suggest that targeted distribution strategies have not led to high community-level coverage nor adequate coverage among the targeted groups. Spatial analysis has revealed significant clustering of households owning a bednet above the underlying population clustering at distances less than 1200 meters. Spatial variables such as distance to a road or a health facility did not fully explain the spatial structure of the data, as indicated by the distribution of the random intercepts for sublocations, indicating there are other spatial or neighborhood determinants not captured in our analysis. Previous studies have not incorporated a point-pattern analysis such as the one here. This analysis highlights the spatial heterogeneity of household bednet coverage and may indicate an inequality in physical, financial, or social access not captured in the set of variables we were able to explore.

In our study, bednet ownership was self-reported which may result in an underestimate of bednet ownership if reported absence of bednets is thought to be linked to receiving a new or additional bednet. We did not distinguish between treated and untreated bednets, which limits our ability to extrapolate the results of our study to predict protection. We did not assess bednet usage and it has been shown that bednet ownership does not predict use [29], [30]. Our analysis of household-level bednet ownership has highlighted variations in ownership and has estimated factors affecting household ownership and population-level coverage. However, the factors explored here explained only a small fraction of the variation in bednet ownership as evidenced by a small R^2^ value. Studies estimating factors affecting household and population-level coverage are critical to evaluating the equity and effectiveness of distribution mechanisms. While the current national malaria strategy has planned for mass distributions every three years, a campaign has not taken place in Bungoma East district in the last five years. Bednet useful life studies have shown rapidly decreasing life after three years, with an average bednet survival of 1–3 years [31]. Further investigations into the impact of frequency and geographic scope of supplemental distribution strategies are therefore critical to achieving adequate coverage to realize population-level indirect effects from bednets.
